# Tradition of wrapping grains in funerary rituals 2200 years ago

**DOI:** 10.1093/nsr/nwag504

**Published:** 2026-08-18

**Authors:** Fan Yang, Yong Ge, Ling Fang, Haiyan Wang, Shuzhi Wang, Jiajun Wang, Zhiguo Zhang

**Affiliations:** National Center for Archaeology, China; Institute of Vertebrate Paleontology and Paleoanthropology, Chinese Academy of Sciences, China; Key Scientific Research Base on Paleolithic Human Evolution and Paleogenetics (IVPP), SACH, China; Anhui Provincial Institute of Cultural Relics and Archaeology, China; School of History, Capital Normal University, China; Key Laboratory of Scientific Archaeology and Cultural Heritage Conservation, Chinese Academy of Social Sciences, China; National Center for Archaeology, China; National Center for Archaeology, China

China has a long and continuous civilization that has fostered numerous traditions. The Dragon Boat Festival is one of China’s most important traditional festivals, celebrated by eating *zongzi*: rice or millet wrapped in leaves. *Zongzi* are traditionally linked to the commemoration of Qu Yuan (c. 340–278 BC), a poet and politician of the Chu Kingdom who, according to legend, drowned himself in the Miluo River. To prevent fish from consuming his body, people threw *zongzi* into the river as offerings. However, the earliest unequivocal evidence of *zongzi* dates only to the Song Dynasty [1,2], leaving its origin and function debated owing to a lack of archaeological evidence.

Here we present well-dated evidence of plant bundles from the Wuwangdun Site, the first well-excavated tomb of King Kaolie of Chu (d. 238 BC) from the Warring States period (Fig. 1 and Supplementary Data: Site information). AMS (Accelerator Mass Spectrometry) ^14^C dating of plant material from the bundles yielded a radiocarbon age of approximately 2200 cal. BP (Table S1 and Fig. S1), placing them chronologically within the late Warring States period and contemporary with the historical context of the Qu Yuan legend. We argue that these bundles represent a funerary practice structurally identical to *zongzi*, thereby bridging the gap between legend and material culture.

**Figure 1.**
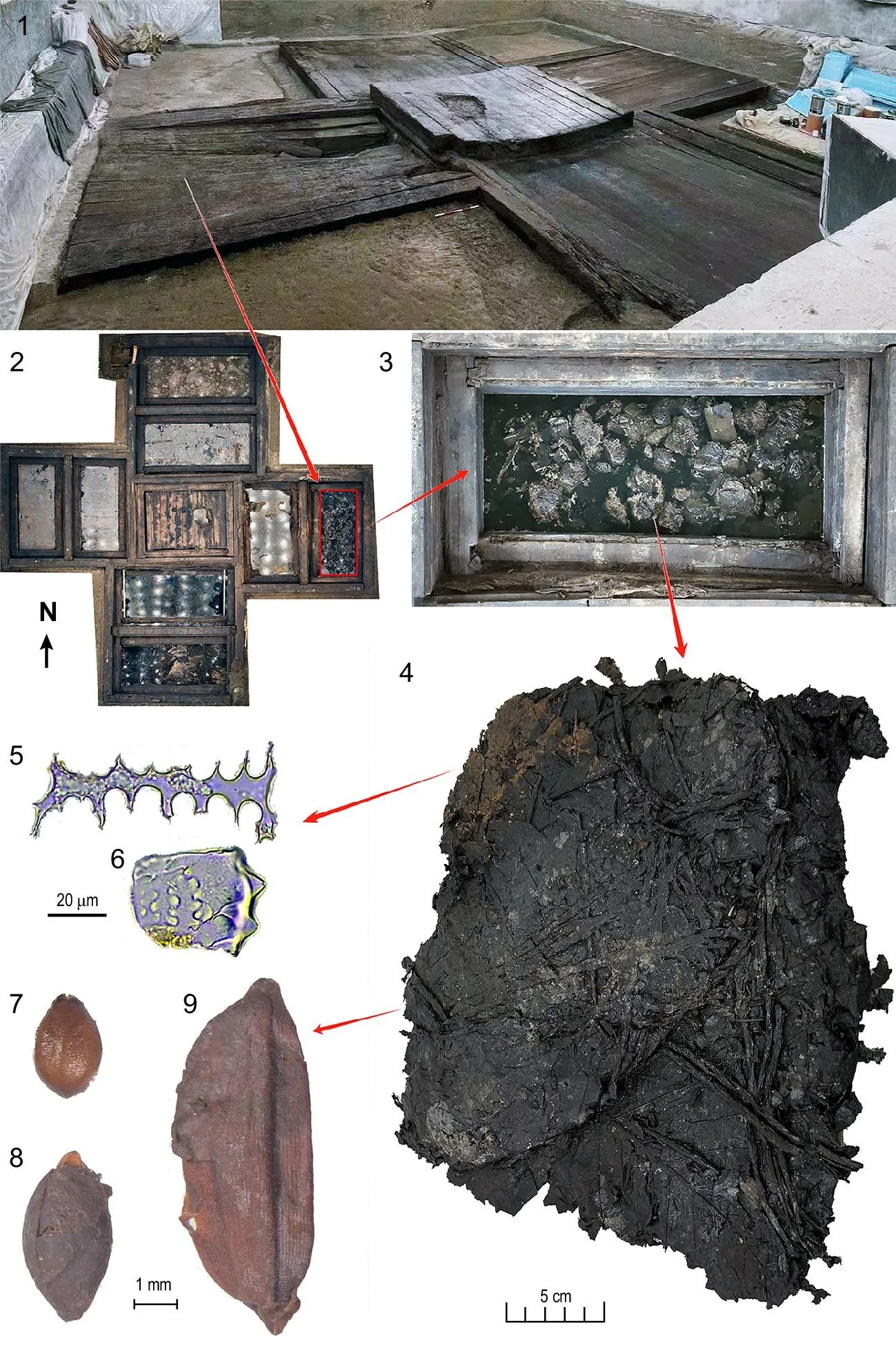
Overview of the Wuwangdun site, plant bundles and main findings. (1) The Wuwangdun excavation site. (2) Top view of Tomb 1 after removal of the cover woods. (3) Waterlogged East I chamber with plant bundles floating everywhere. (4) The cord-wrapped plant bundle, showing brown residue enclosed by the leaves. (5) Elongate dendritic phytolith (cereal type), recovered from the brown residue. (6) Double-peaked phytolith from rice, recovered from the brown residue. (7) *Setaria italica* husk wrapped in leaves. (8) *Panicum miliaceum* husk wrapped in leaves. (9) *Oryza sativa* husk wrapped in leaves.

Several piles of plant bundles were discovered in the East I Chamber of the Wuwangdun Site, well preserved owing to waterlogged conditions. These bundles consist of compact plant material and externally appear to have been wrapped in leaves and tied with cords. After laboratory cleaning, 12 bundles were randomly selected and carefully unwrapped, revealing brown residues and plant seeds. The leaves, cords, residues and seeds were subjected to morphological and microfossil analyses.

Morphological analysis revealed that the leaves are oblong-oblanceolate to obovate, with a short acute apex, a broadly cuneate to subrounded base, and sinuate-dentate margins (Fig. S2). The venation is pinnate and camptodromous, featuring a straight midvein, regularly arranged secondary veins, and tertiary veins perpendicular to the secondaries that are either unbranched or dichotomously branched—a pattern identical to that of modern *Quercus dentata*. This identification was further supported by a comparative analysis of leaf epidermal cells between the archaeological specimens and their modern counterparts.

The binding material was found wrapped around the leaves, securing them in a bundle. Microscopic examination revealed no diagnostic fibres of typical hemp (*Cannabis sativa*), only plant cell and tissue debris, suggesting that the cordage was not made from hemp. Further phytolith analysis of two cord samples identified a large proportion of bilobate and saddle phytoliths derived from the subfamilies Panicoideae and Arundinoideae (Table S2 and Fig. S3). Notably, in cord sample E1-643, some bilobate phytoliths remained articulated, accounting for 25.38% of the total assemblage; in E1-610, some bulliform phytoliths remained articulated, accounting for 35.74% of the total assemblage, showing that these phytoliths originated from the cordage material without severe corrosion. These findings indicate that the cordage may have had multiple origins and was most likely manufactured from plants in the subfamilies Panicoideae and Arundinoideae.

Morphological identification of 456 seeds from 10 bundles revealed that rice (*Oryza sativa*, 43.6%), broomcorn millet (*Panicum miliaceum*, 26.1%) and foxtail millet (*Setaria italica*, 28.5%) dominated the assemblage (Table S3). Most seeds were preserved only as husks (Fig. 1-7, 1-8 and 1-9); a single partially decayed rice grain was recovered. Although glutinous rice and glutinous broomcorn millet are the typical grains used in *zongzi* today, it remains unknown whether the archaeological specimens represent sticky or non-sticky varieties. Other finds included one seed of *Zanthoxylum bungeanum*, one of *Malva* sp. and six of *Scirpus juncoides* (Fig. S4). The first two are aromatic species known as spices, whereas *S. juncoides* is a common rice-paddy weed, suggesting possible admixture during harvesting.

While taphonomic processes may have led to grain decay and preservation bias, the abundance of intact husks nonetheless indicates that the grains were unsuitable—or unintended—for consumption. This pattern is echoed at the Chengyangcheng Site, where whole husks also predominate [3]. Thus, the dominance of husks indicates that the bundles were not intended for consumption by the living. Their presence in a tomb context, however, suggests a funerary function. In early Chinese mortuary practice, grains symbolized agricultural wealth and sustaining power. The Qin wooden slip *Taiyuanyousizhe* (Taiyuan Has a Dead Man, c. late Warring States to Qin dynasty, 3rd century BC) records that the dead valued soybeans as gold and millet as money for paying taxes in the underworld [4]. This indicates that grain held a

symbolic, ritual value beyond its role as food. We therefore interpret these bundles not as provisions for the deceased but as offerings—a functional parallel to the *zongzi* later cast into rivers in memory of Qu Yuan.

Thin layers of brown residue were observed inside the wrapping leaves associated with the grain husks and were provisionally identified as rice or millet flour, given the presence of seeds. Four samples were therefore subjected to microfossil analysis. Starch grain analysis revealed no starch granules, ruling out the possibility that the brown residue was flour or dough made from ground grains. Instead, the residue yielded hundreds of Poaceae pollen grains—with no other pollen types present—indicating a strong association with Poaceae inflorescences. Further phytolith analysis revealed elongate dendritic phytoliths, a type diagnostic of the inflorescence bracts of wheat and barley [5]. Their proportion reached 9.59% in sample E643-2, and ranged from 0.66% to 2.61% in three other samples (Table S2). This finding expands the range of cereals represented in the bundles to include at least four of the ancient ‘five grains’. Because the pollen and phytoliths derive from the non-edible, chaff parts of the inflorescence, their presence—together with the husks of the rice and millet seeds—argues against the interpretation of the bundles as food for direct consumption. We conclude instead that these plant parts were deliberately included as symbolic components of a ritual offering.

Taken together, the plant bundles reveal a consistent wrapping practice: rice, millet and other cereals wrapped in *Q. dentata* leaves and bound with cords made from Poaceae plants. This is consistent with the modern method of making *zongzi*—sticky rice or millet wrapped in leaves and tied with rope. Although these ancient bundles are not identical to modern *zongzi*, they share the same essential characteristics. This practice is also recorded in historical texts. The *Fengtu Ji* (Records of Local Customs and Conditions, c. 236–297 AD), written by Chu Zhou of the Jin Dynasty, states that *zongzi* was made by wrapping sticky rice with millet in *Zizania latifolia* leaves. The *Xu Qixie Ji* (Sequel to the Records of Strange Things, c. 510–520 AD), a collection of supernatural tales by Jun Wu of the Liang Dynasty, records that five-colour ropes were used to bind *zongzi* with *Melia azedarach* leaves to prevent flood dragons from stealing offerings intended for Qu Yuan. These historical accounts corroborate the distinct linkage—leaves wrapping grains bound with cords—observed in the archaeological finds, the legends and modern *zongzi*.

The archaeological findings are contemporary with Qu Yuan and the legends associated with him, both rooted in the Chu Kingdom. Similar bundles from Tomb 8 at the Chengyangcheng Site (c. 2350–2195 years ago) used *Q. dentata* leaves and contained rice and broomcorn millet, with *Z. bungeanum* present in the coffin but not inside the bundles [3]. The consistency in morphology and materials between the Chengyangcheng finds and our study suggests that wrapping grains for funerary rituals was common in the Chu Kingdom during the Warring States period. Given that these plant bundles served as funerary offerings, they align with the legend of *zongzi* being used to commemorate Qu Yuan. Notably, *Q. dentata* leaves are still used today to wrap *zongzi* in parts of Shandong, Shanxi and Henan provinces [6]—regions that once lay along the northern border of the Chu Kingdom—reflecting a culinary tradition that has persisted for over two millennia. Thus, where legend and archaeological evidence converge, these bundles most likely represent a possible precursor of *zongzi*.

Collectively, although the plant bundles represent at most a possible precursor rather than *zongzi* per se, our findings demonstrate that the tradition of wrapping grains for funerary rituals—using plant leaves to contain rice, millet and other cereals, secured with cord—began in the Chu Kingdom no later than 2200 years ago. This tradition was subsequently carried forward and transformed, becoming an enduring element of regional cultural identity.

## Supplementary Material

nwag504_Supplemental_File

## References

[1] Sun J . (in Chinese) Agricult Archaeol 1989; 294–5.

[2] Anhui Provincial Institute of Cultural Relics and Archaeology Commission for Preservation of Ancient Monuments, N.C. (in Chinese) Cult Relics 2016; 34–49.

[3] Lan W . (in Chinese) Huaxia Archaeol 2020; 66–70.

[4] Li L . (in Chinese) Cult Relics 2012; 81–4.

[5] Ball T, Chandler-Ezell K, Dickau R et al. J Archaeol Sci 2016; 68: 32–45. 10.1016/j.jas.2015.08.010

[6] Lin F, Luo B, Long B et al. J Ethnobiol Ethnomed 2019; 15: 63. 10.1186/s13002-019-0339-7 31829257 PMC6907129

