# Supplementary material for: Tradition of wrapping grains in funerary rituals 2200 years ago
Source: Natl Sci Rev. 2026 Aug 18;13(17):nwag504. doi: 10.1093/nsr/nwag504 (PMC13552061; doi:10.1093/nsr/nwag504)
Supplement: Supplementary file 1 [file nwag504_supplemental_file.docx]

Supplementary Information for

Tradition of wrapping grains in funerary rituals 2200 years ago

Fan Yang^1^, Yong Ge^2,3*^, Ling Fang^4^, Haiyan Wang^5^, Shuzhi Wang^6^, Xin Yan^1^, Jiajun Wang^1^, Zhiguo Zhang^1*^

1. National Center for Archaeology, Beijing, 100013, China.

2. Institute of Vertebrate Paleontology and Paleoanthropology, Chinese Academy of Sciences, Beijing, 100044, China

3. Key Scientific Research Base on Paleolithic Human Evolution and Paleogenetics (IVPP), SACH. Beijing, 100044, China.

4. Anhui Provincial Institute of Cultural Relics and Archaeology, Hefei, 230600, China.

5. Capital Normal University, Beijing, 100048, China.

6. Key Laboratory of Scientific Archaeology and Cultural Heritage Conservation, Chinese Academy of Social Sciences, Beijing, 102445, China.

This file includes:

**Site information**

**Material and methods**

**Tables S1 to S3**

**Figs. S1 to S4**

**References**

**Site information**

The main tomb of the Wuwangdun Site (Tomb 1) is located in Xuwa Village, Sanhe Town, Tianjia'an District, Huainan City, Anhui Province (E116°57′01.32″，N32°35′08.00″). It represents the largest, highest-ranking, and most structurally complex King’s tomb of Chu ever discovered through scientific excavation. The Wuwangdun Site is an independent mausoleum centred on the main tomb, comprising the main tomb (Tomb 1), chariot and horse pits, accompanying burials, sacrificial pits, and other features, all enclosed by a moat.

Since 2020, the Anhui Provincial Institute of Cultural Relics and Archaeology, in collaboration with the National Centre for Archaeology (National Cultural Heritage Administration), Shandong University, Xiamen University, and other institutions, has been conducting excavations at the main tomb of the Wuwangdun Site (Tomb 1). The main tomb is a large, "jia"-shaped (甲字形) vertical earthen pit tomb, composed of a sealed mound, a tomb passage, a burial pit, and a chamber structure, featuring a single east-facing sloping ramp. The bottom of the burial pit contains a "ya"-shaped (亞字形) wooden chamber constructed of rectangular timbers. The coffin chamber is located in the centre of the wooden chamber, containing three nested coffins. On each of the four cardinal directions (east, south, west, and north), there are two side chambers (box-type compartments).

The East I Chamber has remained undisturbed. The artifacts unearthed from this chamber are predominantly bronze ritual vessels, with approximately 150 bronze objects, including *ding*- tripods (bronze ritual food vessels), *gui*- food containers (bronze ritual grain vessels), *fu*- vessels (rectangular bronze grain containers), *dui*- vessels (spherical bronze food containers), *fang*- vessels (square bronze wine or water jars), *hu*- jars (bronze wine or water vessels), *yan*- steamers (bronze steaming vessels), *dou*- stemmed vessels (bronze ritual food servers), *jian*- basins (large bronze water vessels), *fu*- cauldrons (bronze cooking vessels), *pan*- plates (bronze water basins), and *zunfou*- wine containers (bronze ritual wine vessels). Among these, two sets of "nine *ding* and eight *gui*" confirm the tomb's royal status and suggest this chamber might have been used to store food-related offerings. The plant bundles in this chamber were selected for this study. Regardless of the plant bundles, animal bones were also observed spread in the chamber due to the waterlogged environment.

Regarding the identity of the tomb occupant, an inscription was found on the mouth rim of a bronze *fu*, reading: "chu wang Yan Qian zuo zhu jin fu yi gong sui chang". The name "Yan Qian" can be interpreted as "Xiong Wan." Based on a comprehensive analysis of historical records and excavated written materials, the tomb owner is identified as King Kaolie of Chu (Xiong Yuan), as recorded in the Records of the Grand Historian Hereditary House of Chu.

This region has a long history of rice, millets and cereal cultivation and consumption, as evidenced by both archaeobotanical and textual records. Archaeobotanical studies have confirmed that rice dominated this region prior to the Chu Kingdom (Zhao, 2020), while millets and other cereals were gradually introduced during the Dawenkou Culture period and steadily increased in importance over time (Zhao, 2022). Thus, the rice, foxtail millet, broomcorn millet and wheat recovered from the plant bundles correspond to the staple crops cultivated and consumed in the Chu Kingdom. This agricultural picture is further corroborated by contemporary textual records. Slip No. 2-029 from the bamboo manuscripts of Tomb No. 1 at Changtaiguan records "two *fu* vessels filled with fine foxtail millet" (Chen, 2009). The character *liang* (粱) refers to superior-quality foxtail millet, while *fu* was a ritual vessel specifically used to hold grain offerings during sacrifices. This evidence points to the prominent status of foxtail millet in the Chu dietary tradition. Additional support comes from the *Zhao Hun* (Summons of the Soul) in the *Chu Ci*, which states: "Rice, foxtail millet, early-ripening wheat and sticky broomcorn millet were mixed together and cooked" (Hong and Bai, 1983). This annotation confirms that these grains were widely cultivated and consumed in the Chu Kingdom during the Warring States period.

**Material and methods**

After opening the wooden lid of the East I Chamber, a large number of plant bundles were revealed. Some of these bundles were floating on the water surface within the chamber, while others became scattered across various locations in the East I Chamber as the standing water was drained. During the process of cleaning the East I Chamber, all plant bundles were gradually extracted and brought back to the laboratory, totalling 180 bundles. Subsequently, 10 plant bundles were randomly selected for layer-by-layer unpacking, and 2 were used for microfossil analysis. Visible plant seeds were picked during the unpacking of the plant bundles, preserved in deionised water, and identified under a Leica M165 stereomicroscope.

Plant materials from the plant bundles, including the leaves, the husks of seeds, and the cords from the plant bundles E1:11, were sent to the Radiocarbon Dating Laboratory, School of Archaeology and Museology, Peking University, for AMS (Accelerator Mass Spectrometry) ^14^C dating. The plant samples were pretreated using the acid-base-acid (A-B-A) method (Southon & Magana, 2010), followed by graphite synthesis via the vacuum-line hydrogen method, prior to accelerator mass spectrometry measurements.

The leaves and cords were sent to the Laboratory of Wood Archaeology, Key Laboratory of Scientific Archaeology and Cultural Heritage Preservation, Chinese Academy of Social Sciences, for species identification. The plant remains picked out from the bundles were brought back to the Plant Archaeology Laboratory of the National Centre for Archaeology (National Cultural Heritage Administration) for identification, measurement, and photographing using a Leica M165 stereomicroscope and a Leica DM 750 microscope. For leaf morphological identification, we selected several well-preserved specimens with intact leaf blades and relatively clear venation patterns. In addition, modern leaves of *Quercus aliena*, *Quercus acutissima*, and *Quercus dentata* were collected for comparative analysis, and identification was also assisted with reference photographs from the Plant Photo Bank of China (PPBC). After leaf morphological examination, epidermal cells were also extracted using the wet ashing method: samples were treated with 10% hydrochloric acid to remove carbonates, and then digested in concentrated nitric acid in a hot water bath. Epidermal cells were separated by heavy liquid flotation (ZnBr_2_, 2.0 g/cm^3^). After centrifugal washing with distilled water to remove the heavy liquid, the epidermal cells were mounted on slides and observed under a microscope.

The residues and cords were sent to the Institute of Vertebrate Paleontology and Paleoanthropology, Chinese Academy of Sciences, for microfossil analysis. During sampling, we selected areas that were well wrapped by leaves and in the centre parts to avoid environmental contamination. 1g of fresh samples collected from the brown residues, as well as a 2cm-long cord in E610 and E643, was transferred to centrifuge tubes for microfossil extraction. The extraction of starch grain followed the published methods by Yang et al. 2012, the extraction of phytolith from residues followed the published methods by Zhang et al. 2024, and the extraction of phytolith from cords followed the published methods by Ge et al. 2022. The extracted microfossils were observed under a Leica DM 750 microscope.

Table S1 AMS ^14^C dating results of the plant materials

| **Lab code** | **Original code** | **Material** | **Place** | **Proveniece** | **Original dating（BP）** | **Cal BC** | **Cal BC** |
| --- | --- | --- | --- | --- | --- | --- | --- |
|  |  |  |  |  |  | **1σ (68.3%)** | **2σ (95.4%)** |
| BA240760 | 3 | Husks of *Setaria italica* | Huainan Anhui | Chamber East I | 2215±20 | 358BC (8.4%) 346BC  314BC (28.0%) 276BC  260BC (9.9%) 244BC  234BC (22.0%) 204BC | 372BC (15.5%) 338BC  325BC (79.9%) 198BC |
| BA240763 | 6 | Husks of *Oryza sativa* | Huainan Anhui | Chamber East I | 2175±20 | 349BC (38.7%) 310BC  206BC (29.6%) 174BC | 356BC (53.0%) 278BC  257BC (1.1%) 248BC  232BC (41.3%) 158BC |
| BA240764 | 7 | Leaves of *Quercus dentata* | Huainan Anhui | Chamber East I | 2175±20 | 349BC (38.7%) 310BC  206BC (29.6%) 174BC | 356BC (53.0%) 278BC  257BC (1.1%) 248BC  232BC (41.3%) 158BC |
| BA240765 | 8 | cords | Huainan Anhui | Chamber East I | 2190±20 | 353BC (50.2%) 286BC  228BC (7.2%) 216BC  212BC (10.9%) 197BC | 359BC (57.2%) 275BC  262BC (4.7%) 244BC  234BC (33.6%) 172BC |

Notes: the calibration curve used is the IntCal20 Curve (Reimer et al., 2020), and the program employed is OxCal v4.4.4 (Bronk Ramsey, 2009; 2021). Abbreviations: BC (Before Christ), BP (Before Present, before the year 1950), σ (sigma, Standard Deviation).

Table S2 Phytolith assemblages (%) from the brown residue and cords

| Sample | Elongate entire | Acute | Rondel | Bilobate | Saddle | Bulliform flabellate | Bulliform blocky | Elongate dendritic | Double-peaked | Amoeboid branchiate | Total count |
| --- | --- | --- | --- | --- | --- | --- | --- | --- | --- | --- | --- |
| E1 610-2 | 22.88 | 9.80 | 3.92 | 14.38 | 9.15 | 15.69 | 20.26 | 2.61 | 0.00 | 1.31 | 306 |
| E1 610-4 | 25.81 | 7.26 | 3.23 | 10.48 | 8.06 | 21.77 | 17.74 | 2.42 | 0.00 | 3.23 | 248 |
| E1 643-1 | 22.52 | 6.29 | 2.65 | 15.56 | 14.24 | 20.53 | 17.22 | 0.66 | 0.00 | 0.33 | 302 |
| E1 643-2 | 22.60 | 3.42 | 2.74 | 8.22 | 10.27 | 22.95 | 19.86 | 9.59 | 0.34 | 0.00 | 292 |
| E1 610-cord | 8.84 | 0.40 | 1.20 | 5.62 | 12.85 | 35.74 | 35.34 | 0.00 | 0.00 | 0.00 | 249 |
| E1 643-cord | 7.31 | 0.77 | 0.77 | 25.38 | 14.62 | 16.92 | 34.23 | 0.00 | 0.00 | 0.00 | 260 |

Table S3 Number of seeds recovered from the studied plant bundles

| Plant bundle code | *Setaria italica* | *Panicum mileaceum* | *Oryza sativa* | *Malva* sp. | *Zanthoxylum bungeanum* | *Scirpus juncoides* |
| --- | --- | --- | --- | --- | --- | --- |
| E1：202 | 21 | 15 | 29 | 1 | 1 | 0 |
| E1：11 | 32 | 21 | 36 | 0 | 0 | 2 |
| E1：22 | 9 | 11 | 15 | 0 | 0 | 0 |
| E1：57 | 12 | 9 | 17 | 0 | 0 | 2 |
| E1：56 | 13 | 7 | 15 | 0 | 0 | 0 |
| E1：58 | 10 | 15 | 19 | 0 | 0 | 0 |
| E1：287 | 19 | 10 | 20 | 0 | 0 | 0 |
| E1：293 | 0 | 11 | 20 | 0 | 0 | 0 |
| E1：507 | 8 | 12 | 13 | 0 | 0 | 2 |
| E1：489 | 6 | 8 | 15 | 0 | 0 | 0 |
| Total number | 130 | 119 | 199 | 1 | 1 | 6 |

Figure S1. Distribution of the AMS ^14^C dates

Figure S2. Morphology of the archaeological leaf specimen (1) and its margin (2), stomata and epidermal cells of the archaeological specimen (3) and modern *Quercus dentata* (4).

Figure S3 Atlas of microfossils recovered from the brown residue and cords (left) and modern reference of saddle phytoliths (right). Left: 1. Bulliform flabellate, 2. Bulliform blocky, 3. Double-peaked, 4. Elongate dendritic, 5. Acute, 6. Elongate entire, 7. Rondel, 8. Saddle, 9. Amoeboid branchiate, originated from ferns, 10. Poaceae pollen grain from the brown residue, 11. In *situ* bilobate phytoliths from the cord, 12. In *situ* bulliform blocky from the cord. Right: 1. Saddle phytolith from Chloridoideae, 2. Saddle phytolith from Arundinoideae, 3. Saddle phytolith from Bambusoideae.

Figure S4. Atlas of seeds recovered from the plant bundles. 1. *Setaria italica*, 2. *Malva sp.*, 3. *Scirpus juncoides*, 4. *Panicum miliaceum*, 5. *Zanthoxylum bungeanum*, 6. *Rice grain*, 7. *Oryza sativa*.

**References**

Bronk Ramsey C. Bayesian Analysis of Radiocarbon Dates[J]. Radiocarbon, 2009, 51(1): 337–360.

Bronk Ramsey C. OxCal v4.4.4[CP/OL]. Oxford: Oxford Radiocarbon Accelerator Unit, University of Oxford, 2021. <https://c14.arch.ox.ac.uk/oxcal.html>.

Chen W (Eds.). Fourteen Sets of Warring States Bamboo Slips Excavated from Chu Regions. Beijing: Economic Science Press, 2009: 384.

Ge, Y., Jin Y., and Zhang X., Phytolith Production and Morphotypes in Modern Plants on the Tibetan Plateau. Front Plant Sci, 2022. 13: p. 950322.

Hong, X. Z. (Song Dynasty), & Bai, H. W. et al. (Eds.). Chu Ci Bu Zhu (Annotations on Chu Ci with Supplementary Notes). Beijing: Zhonghua Book Company, 1983, p. 101.

Plant Photo Bank of China, <https://ppbc.iplant.cn/>, visited by 2026-04-10, reference id: 5945763, 4793209, 18996918.

Reimer P J et al. The IntCal20 Northern Hemisphere Radiocarbon Age Calibration Curve (0–55 cal kBP). Radiocarbon, 2020, 62(4): 725–757.

Southon, J.R., & Magana, A.L. (2010). A comparison of cellulose extraction and ABA pretreatment methods for AMS 14C dating of ancient wood. Radiocarbon, 52(3), 1371–1379.

Yang, X., et al., Early millet use in northern China. Proc Natl Acad Sci U S A, 2012. 109(10): p. 3726–30.

Zhang, J., et al., Rice’s trajectory from wild to domesticated in East Asia. Science, 2024, 384(6698): p. 901–906.

Zhao Z., Origin of Agriculture and Archaeobotanical Works in China. Agricultural History of China, 2020, (4): p. 3-9.

Zhao, Z. Z., A study of agriculture from Longshan Period to the Zhou Dynasty in the middle Huai River Valley, 2022, [Doctoral dissertation, Shandong University].
